# Computational Study on the Effect of Inactivating/Activating Mutations on the Inhibition of MEK1 by Trametinib

**DOI:** 10.3390/ijms21062167

**Published:** 2020-03-21

**Authors:** Jingxuan Zhu, Congcong Li, Hengzheng Yang, Xiaoqing Guo, Tianci Huang, Weiwei Han

**Affiliations:** Key Laboratory for Molecular Enzymology and Engineering of Ministry of Education, School of Life Science, Jilin University, 2699 Qianjin Street, Changchun 130012, China; zhujx15@mails.jlu.edu.cn (J.Z.); licongcong097@163.com (C.L.); yanghz1317@mails.jlu.edu.cn (H.Y.); guoxq1316@mails.jlu.edu.cn (X.G.); 17843123225@163.com (T.H.)

**Keywords:** MEK1, trametinib, docking, molecular dynamic simulations, steered molecular dynamic simulations, allosteric channel

## Abstract

Activation of the mitogen-activated protein kinase (MAPK) signaling pathway regulated by human MAP kinase 1 (MEK1) is associated with the carcinogenesis and progression of numerous cancers. In addition, two active mutations (P124S and E203K) have been reported to enhance the activity of MEK1, thereby eventually leading to the tumorigenesis of cancer. Trametinib is an MEK1 inhibitor for treating EML4-ALK-positive, EGFR-activated, and KRAS-mutant lung cancers. Therefore, in this study, molecular docking and molecular dynamic (MD) simulations were performed to explore the effects of inactive/active mutations (A52V/P124S and E203K) on the conformational changes of MEK1 and the changes in the interaction of MEK1 with trametinib. Moreover, steered molecular dynamic (SMD) simulations were further utilized to compare the dissociation processes of trametinib from the wild-type (WT) MEK1 and two active mutants (P124S and E203K). As a result, trametinib had stronger interactions with the non-active MEK1 (WT and A52V mutant) than the two active mutants (P124S and E203K). Moreover, two active mutants may make the allosteric channel of MEK1 wider and shorter than that of the non-active types (WT and A52V mutant). Hence, trametinib could dissociate from the active mutants (P124S and E203K) more easily compared with the WT MEK1. In summary, our theoretical results demonstrated that the active mutations may attenuate the inhibitory effects of MEK inhibitor (trametinib) on MEK1, which could be crucial clues for future anti-cancer treatment.

## 1. Introduction

Mitogen-activated protein kinase kinase (MAPKK, also known as MEK) occupies a crucial signaling node in the RAS-RAF-MEK-ERK mitogen-activated protein kinase (MAPK) signaling cascade. Among them, RAF kinases are vitally involved in the phosphorylation and activation of MEK. There are two forms of MEK in humans, including MEK1 and MEK2, which share 79% identical amino acid sequence and have equal ability to phosphorylate specific tyrosine and threonine residues for the downstream target ERK substrates [1,2,3]. Enhanced MEK activity can cause abnormal activation in the RAS-RAF-MEK-ERK pathway, which has been reported to be responsible for the pathogenesis of inflammation and approximately 30% of all human malignancies [4,5]. Thus, MEK has been the target of various drug discoveries [6,7,8,9,10,11,12,13,14], and inhibition of MEK activity may be used to treat MEK pathway activation-driven cancers.

**Figure 1:**
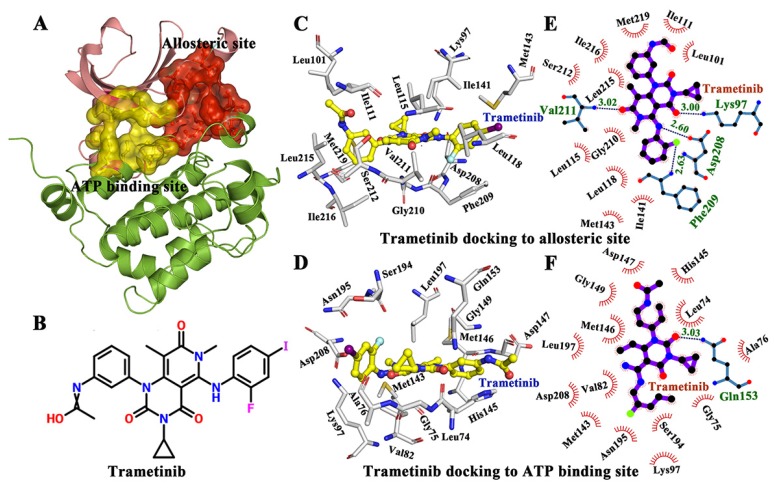
The interactions between protein and ligand in allosteric and ATP binding pockets respectively. (**A**) The allostric binding site and ATP binding site in MAP kinase 1 (MEK1) (PDB Id 3SLS). (**B**) The 2D structure of tramctinib. (**C**) Tramctinib docked to the allosteric site. (**D**) Tramctinib docked to the ATP site. (**E**) The active residues around tramctinib binding to the allosteric site. (**F**) The active residues around tramctinib binding to the ATP site.

In consideration of the critical roles of the MAPK signaling cascade in the pathogenesis of various diseases, it is urgent to identify novel MEK1 inhibitors. MEK inhibitors are synthetic chemicals or drugs that inhibit MEK1 and MEK2 [15], thereby affecting the MAPK/ERK pathway which is generally overactive in certain types of malignancies. Two different binding sites exist in MEK for ligand binding. To be specific, one is called allosteric binding site which can inhibit MEK1 and MEK2, and the other is called ATP binding site (shown in Figure 1A). The allosteric binding site is spatially proximal to the ATP binding site. MEK inhibitors, including trametinib (the 2D structure is shown in Figure 1B) [16,17,18], selumetinib [19] and binimetinib, have been used as a rational multi-therapy strategy to treat RAS-RAF-MEK-ERK pathway-related cancers.

However, the X-ray crystal structure of trametinib bound to MEK1 has not been uncovered to date. Trametinib and cobimetinib are type III allosteric inhibitors of MEK1 because they can bind adjacent to the ATP binding pocket [20,21]. In addition, they are steady-state non-competitive inhibitors with respect to ATP, since ATP cannot prevent their interaction with MEK1. Previous studies have reported the mutations of P124S [22,23,24] and E203K [25,26] in MEK1, which can enhance the catalytic activity of MEK1, thereby increasing the phosphorylation of MEK1 and promoting tumorigenesis. While some mutations (such as A52V) have identified are not activating in vitro, since A52 does not interact strongly with the kinase domain in the wild-type 3D structure [25]. However, the effects of mutations in different regions of MEK1 on the spatial structure of MEK1 remain unknown. To this end, herein, in the present study, docking study was used to identify the binding pose of trametinib to MEK1 (PDB ID 3SLS) [27]. In addition, molecular dynamic (MD) simulations were performed to explore the conformational changes of wild-type (WT) MEK1 and three mutants (A52V, P124S, and E203K) when trametinib bound to the allosteric site. Finally, steered molecular dynamic (SMD) simulations were conducted to investigate the dissociation of trametinib from WT MEK1 and three mutants (A52V, P124S, and E203K). Hopefully, our results can provide some useful clues for designing new inhibitors for the RAS-RAF-MEK-ERK pathway.

## 2. Results and Discussion

### 2.1. Binding Mode of Trametinib to MEK1

Previous studies have shown that trametinib is a noncompetitive inhibitor of MEK1. In this study, a docking study was conducted to compare the binding mode and energy of trametinib to the ATP pocket and allosteric site of MEK1. As a result, the ligand (trametinib) was docked by AutoDock 4.2 [28,29]. The lowest conformations of MEK1-trametinib complex were selected for further docking analysis (Figure 1C,D). In addition, the trametinib bound to the allosteric site of MEK1 had a lower energy score (−5.43 kcal/mol) than that bound to the ATP site (−3.65 kcal/mol), indicating that the allosteric site was optimal for trametinib binding (the lower energy score suggested the stability of the complex). Afterwards, LIGPLOT was used to analyze the interactions between trametinib and two binding sites (ATP and allosteric sites) [30]. As shown in Figure 1E,F, the interactions were mainly mediated by hydrogen bonds and hydrophobic contacts. Particularly, Lys97 (3.00 Å), Asp208 (2.60 Å), Phe209 (2.63 Å), and Val211 (3.02 Å) formed hydrogen bonds with trametinib in the allosteric site, to further facilitate in trametinib binding. Besides, as shown in Figure 1D,F, only Ala76 formed a hydrogen bond with trametinib in ATP binding site. Trametinib was obviously prone to bind to the allosteric site than the ATP site. Additional hydrogen bonds may also be considered to be useful in trametinib binding. Moreover, residues Lys97, Asp208, Phe209, Phe209, and Val211 played important roles in the binding between trametinib and the allosteric site.

### 2.2. Conformational Changes for Trametinib Bound to the WT MEK1 and Three Mutants

**Figure 2:**
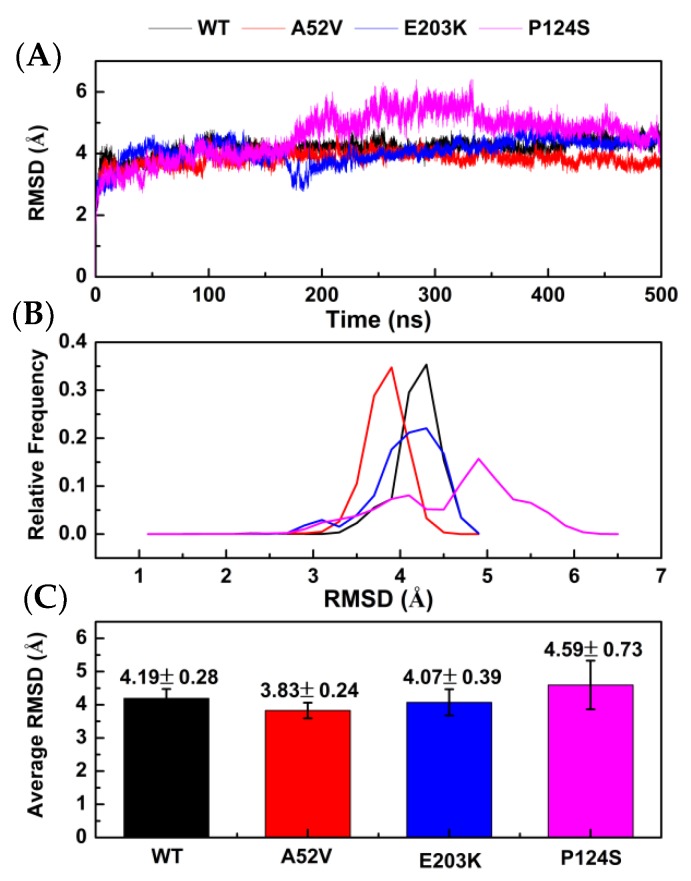
Stability analysis for WT MEK1 and three mutants. (**A**) The root-mean-square deviation (RMSD) plot during 500 ns MD simulations, (**B**) the relative frequency of RMSD plot, (**C**) the average RMSD value.

In present study, 500 ns MD simulations were performed under four systems (WT MEK1–trametinib, A52V MEK1–trametinib, E203K MEK1–trametinib, and P124S MEK1–trametinib) (generated by AutoDock 4.2). The dynamics-based analysis of structural stability was subsequently adopted for the complexes of WT MEK1 and three mutants. First, atom positional root-mean-square deviation (RMSD) of the protein backbone was calculated to evaluate the stability of MD simulations. As shown in Figure 2A, the RMSD values of the four systems were stable around 350 ns. As shown in Figure 2B,C, the average RMSD of trametinib bound to the WT MEK1 and three mutants (A52V, E203K and P124S) was approximately 4.19 ± 0.28 Ǻ, 3.83 ± 0.24 Ǻ, 4.07 ± 0.39 Ǻ, and 4.59 ± 0.73 Ǻ, respectively, indicating that the structures of the four systems already reached relative equilibrium.

**Figure 3:**
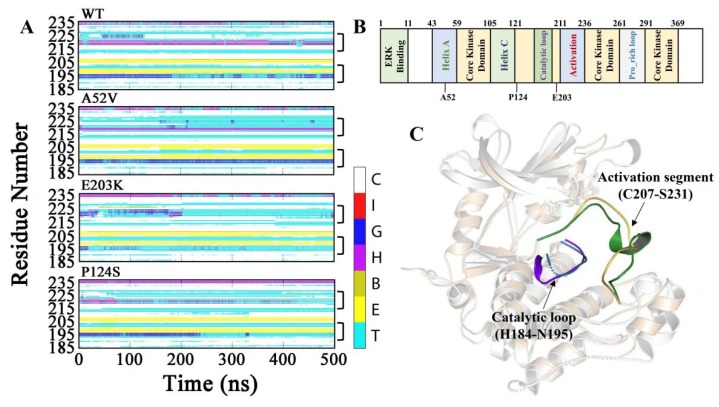
Dynamic changes of the secondary structure profile for (**A**) Activation segment and catalytic loop of WT and mutant MEK1 throughout the simulation. The color bar represented different secondary structures as follow: coil [C], π_helix [I], 3_10__helix [G], α_helix [H], β_Bridge [B], β_bugle [E], turn [T]. (**B**) Functional classification of MEK1 sequence. (**C**) The 3D structure of MEK1(PDB code 3SLS) with highlighted activation segment and catalytic loop.

Subsequently, to explore the conformational changes for the complexes of WT MEK1 and three mutants during the simulations, the secondary structure contents were analyzed (shown in Figure 3A–C, Figure S1 and Table 1). As a result, α_helix content of the activation segment (C207–S231) almost disappeared in the two active mutants (E203K and P124S). Similarly, the content of 3_10__helix in the catalytic loop (H184–N195) was also drastically decreased in the active mutants, compared with that in WT and A52V MEK1. As shown in Table 1, the probability of α_helix and 3_10__helix for residues N214-I216 in the activation segment and P193-N195 in the catalytic loop had an obviously lower score in the active form (E203K and P124S) than that of in the non-active form (WT and A52V). The helix disappearance could give rise to disordered structure in the two-key domain (activation segment and catalytic loop) for E203K and P124S MEK1, which may affect the binding ability of trametinib, thereby regulating the binding of substrate (ATP) to ATP pocket.

**Figure 4:**
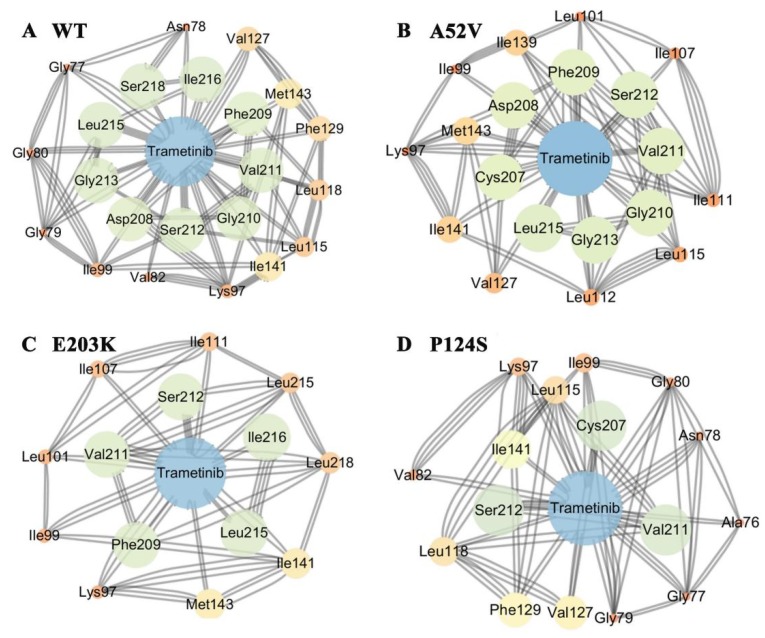
The subnetwork analysis of protein-ligand interaction. The subnetwork between protein and trametinib in (**A**) WT, (**B**) A52V mutant, (**C**) E203K mutant and (**D**) P124S mutant MEK1.

To further explore the effects of the disappearance of two helixes on the non-active and active form for trametinib binding, structure-based network analysis (Figure 4A–D) was carried out to describe the stable interaction communities between the WT MEK1/mutants and trametinib. Consequently, network analysis showed that trametinib could interact with MEK1 via residues at the allosteric site. Compared with the nonactive MEK1, the number of residues in allosteric site associated with trametinib decreased rapidly in the active mutations (shown in Figure 4A–D). Network analysis further illustrated that the active mutants destroyed the interactions between MEK1 and trametinib, causing attenuated inhibitory effects of trametinib on MEK1. The probability of hydrogen bonds formation between the active/nonactive MEK1 and trametinib was summarized in Table 2, which was decreased during 500 ns MD simulations, indicating that trametinib had more potent interactions with the non-active MEK1 (WT and A52V mutant) than that with the active types (P124S and E203K mutant).

### 2.3. Structural Motion of the WT and Mutants MEK1 after Binding with Trametinib

**Figure 5:**
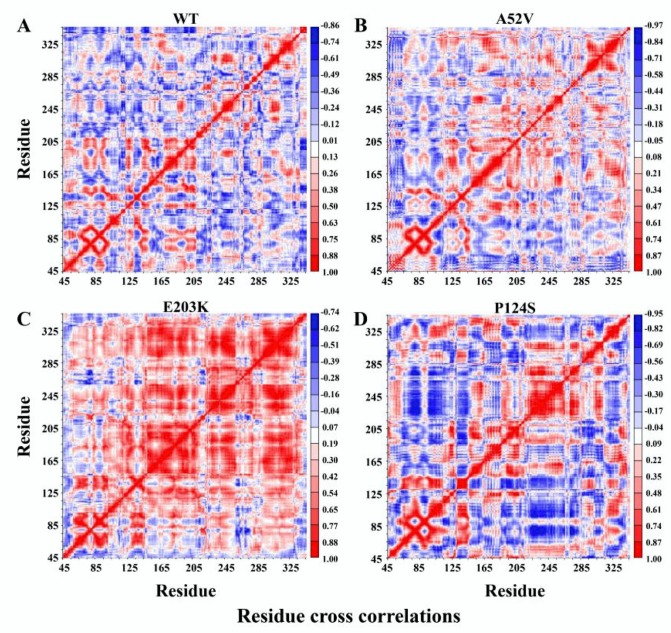
Dynamical cross-correlation map (DCCM) for the 500 ns molecular dynamic (MD) trajectories of (**A**) WT, (**B**) A52V mutant, (**C**) E203K mutant and (**D**) P124S mutant MEK1. Positive regions (colored in red) indicated strongly correlated residue motions, whereas negative regions (colored in blue) indicated anti-correlated movements.

The covariance matrix maps of the four complexes are illustrated in Figure 5A–D, wherein the anti-harmonic and large-scale motions were highlighted by diagonalizing the matrix. The positive regions (red) indicated the strongly correlated motions of residues, while the negative regions (blue) were associated with the anti-correlated movements. Notably, trametinib in the active forms (E203K and P124S mutants) led to strong centralized self-correlated motion (shown in Figure 5C,D). In particular, the activation segment (residues 207–231) exhibited strong fluctuations in the active form (P124S mutant) with trametinib (Figure 5D).

**Figure 6:**
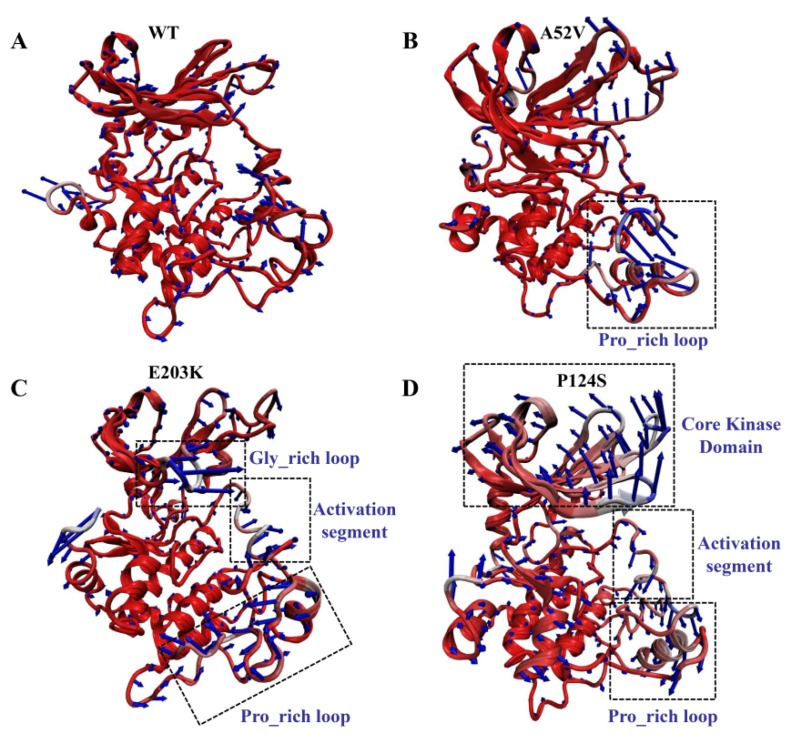
Motions of WT and mutant MEK1 based on the first PC for (**A**) WT, (**B**) A52V mutant, (**C**) E203K mutant and (**D**) P124S mutant MEK1. The arrows represent the directions and amplitudes of the movements.

Despite the elucidation of the generally correlated movements by the dynamical cross-correlation map (DCCM) analysis, the specific motion trend of each region remained ambiguous. Therefore, the extreme projections for the first principal component (PC1) were visualized by presenting the mode direction for each residue with arrows (Figure 6), which described the dominant motions during the 500 ns MD simulation. Observation of PC1 in the WT complex indicated a slight movement, which could maintain the integrity and compactness of MEK1 during the simulation. Similarly, PC1 results in the A52V mutant MEK1, indicating a small amplitude except for the Pro_rich loop (residues 261–291) with moderate displacement (Figure 6A,B). Conversely, the motion of the recognition loop was much stronger in the active mutants (E203K and P124S, Figure 6C,D) than in the WT and non-active mutant MEK1, which was consistent with the flexibility analysis above. For the E203K MEK1, the large movements were concentrated on the Gly_rich loop, activation segment (residues 207–231) and Pro_rich loop (residues 261–291). In terms of the P124S MEK1, the core kinase domain and activation segment showed the opposite movement, which should be responsible for the decreased interactions between MEK1 and trametinib. These motions of domain recognition might be associated with the depressed inhibitory effect of MEK1.

The binding free energy from MM/PBSA calculations can provide a semi-quantitative estimate of inhibitor affinity with protein. The binding free energy of trametinib with WT and mutant MEK1 was listed in Table 3, which revealed that the binding free energy (∆*G*_bind_) of the four systems was negative, indicating that trametinib was energetically favorable in the four systems. Compared with the four binding free energies, the energies between trametinib and the active types (P124S and E203K) were higher than those between trametinib and the non-active types (WT and A52V). These results suggested that trametinib in the non-active types had a higher probable binding energy and pose. Of note, our results were consistent with the experimental data. For binding free energies in each component of MM/PBSA binding free energies, electrostatic energies (∆*E*_ele_) contributed to the total energies to a greater extent than van der Waals energies (∆*E*_vdW_) in the four systems. Thus, electrostatic interaction was considered as the dominant position during the interaction of trametinib with WT and mutant MEK1. In summary, the binding of trametinib to the nonactive types (WT and A52V) can induce more potent interactions than binding to the active forms.

### 2.4. CAVER 3.0 Identifies the Allosteric Channel of MEK1

**Figure 7:**
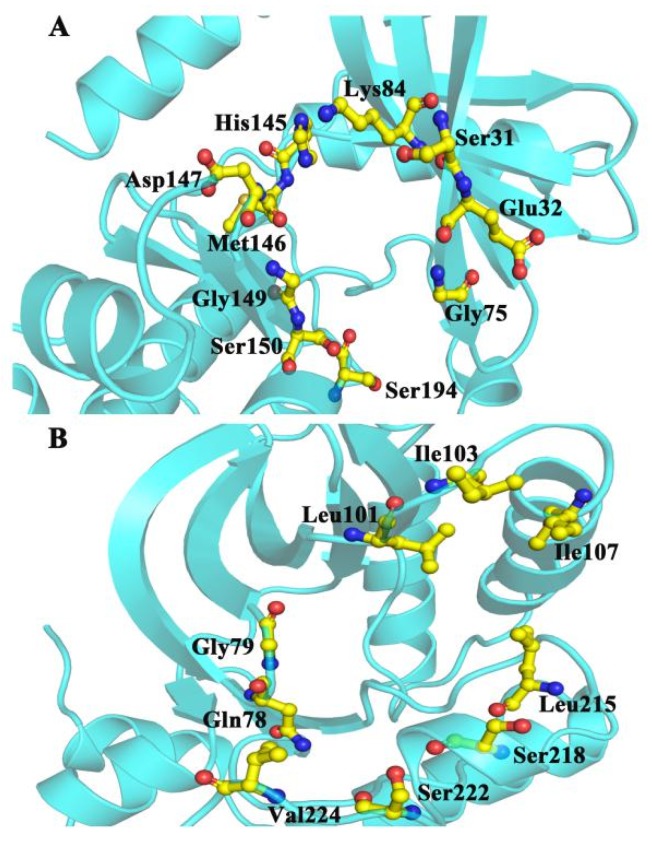
The surrounding residues for (**A**) ATP and (**B**) allosteric channels.

**Figure 8:**
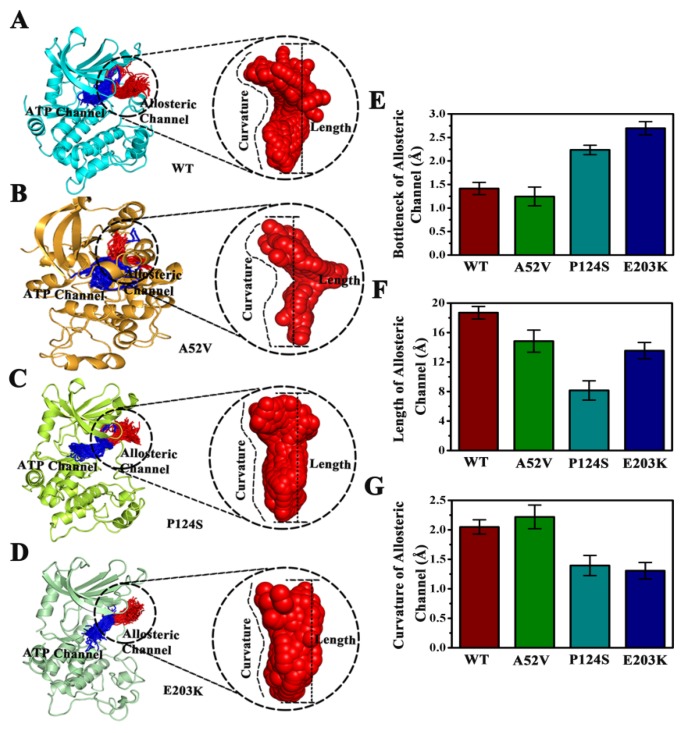
Comparison of the channels about WT, A52V, P124S and E203K complexes. (**A**–**D**) The top ranked collective allosteric channels identified by CAVER 3.0 according to the 500 ns MD simulations trajectories. (**E**) The bottleneck of allosteric channel. (**F**) The length of allosteric channel. (**G**) The curvature of allosteric channel.

CAVER 3.0 was performed to analyze 2500 snapshots from a 500 ns MD simulation of the five systems, which identified the ATP channel and allosteric channel (Figure 7). The ATP channel was lined by the residues Lys84, Ser31, Glu32, Gly75, Ser194, Ser150, Gly149, Met146, Asp147 and His145 (Figure 7A), whereas the allosteric channel was surrounded by Gly79, Glu78, Val224, Ser222, Ser218, Leu215, Ile107, and Ile103 (Figure 7B). The curvature and length of the allosteric channel of the active type (E203K and P124S) and nonactive type (WT and A52V) were shown in Figure 8A–D. In addition, the bottleneck, length, and curvature of the allosteric channel of the active and nonactive type were listed in Figure 8E–G. Taken together, these results suggested that the allosteric channel was wider in active mutants than that of the WT MEK1.

**Figure 9:**
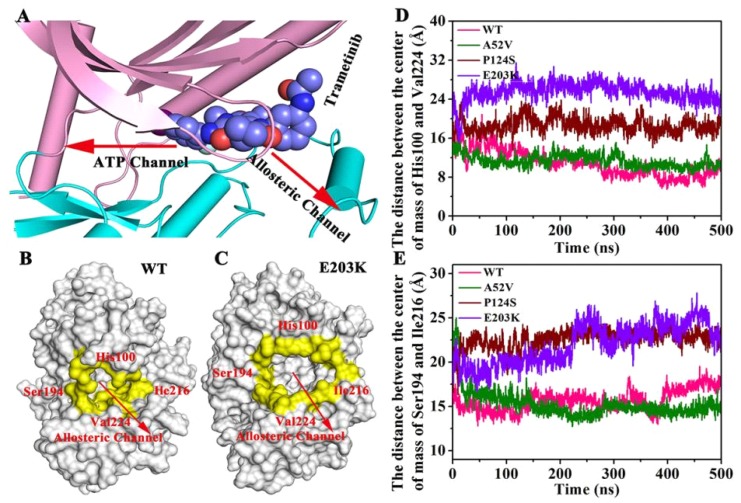
The changes of the active mutations’ mode. (**A**) The directions of allosteric channel and ATP channel. (**B**,**C**) The four residues located at the allosteric channel and faced toward each other. (**D**) The distance between the center of mass of His100 and Val224. (**E**) The distance between the center of mass of Ser194 and Ile216.

In order to identify the changes of the active mutations made (Figure 9A–C), we further measured the distance between the center of mass of His100 and Val224 (Figure 9D) and the distance between the center of mass of Ser194 and Ile216 (Figure 9E). The four residues were located at the allosteric channel and faced towards each other (shown in Figure 9A–C). As shown in Figure 9D,E, the distance either in Ser194 and Ile216 or in His100 and Val224 in the active type (E203K and P124S) was longer than that of in the WT and A52V. That is to say, the curvature of the entrance of allosteric channel in active type was larger than that in nonactive type, which could affect the binding abilities of trametinib to the allosteric site.

### 2.5. Dissociation of Trametinib from the WT MEK1 and Its Three Mutants

**Figure 10:**
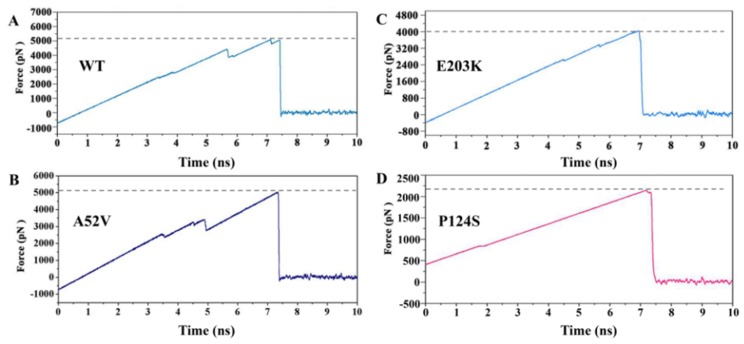
The dissociation process of trametinib from (**A**) WT, (**B**) A52V, (**C**) E203K and (**D**) P124S MEK1.

In our research, SMD simulations were further performed to induce the dissociation of trametinib from the active types (P124S and E203K mutant) and the non-active types (WT and A52V mutant). Each SMD simulation was performed in triplicate (Figure S2). As shown in Figure 10, the pulling force which exerted on trametinib through the active and non-active MEK1 was linearly increased in the initial stage of the SMD simulation, which reached peak at around 7 ns. Finally, the force plunged to zero soon after 7 ns, indicating that the trametinib completely escaped from MEK1. Except for the same points, intriguingly, the values of the pulling force at peak for WT and A52V mutant (around 5123 pN and 5047 pN) were larger than those for P124S and E203K mutant (around 4043 pN and 2192 pN). The above results indicated that the dissociation of trametinib from non-active MEK1 was much easier than that from active MEK1, which were consistent with the above channel analysis. In other words, larger allosteric channel entrance may be responsible for the easy unbinding of trametinib, which would subsequently decrease the inhibitory effect.

## 3. Methods

### 3.1. Preparation of the Protein Structures

The crystal structure (PDB ID: 3SLS) [26] of MEK1 was retrieved from the Protein Data Bank (www.rcsb.org). The structures of three other mutants (P124S, E203K, and A52V) were visualized by SWISS-MODEL (https://swissmodel.expasy.org/) on line software, using WT structure as the template. The inhibitor trametinib was downloaded from Chemspider, followed by optimization using Gaussian 09 software and B3LYP 6031G* set.

### 3.2. Docking Study

AutoDock 4.2 was used for docking, and the Lamarckian genetic algorithm in AutoDock 4.2 program [31,32,33] was employed to identify the appropriate binding modes and the conformation of trametinib to MEK1. Moreover, the grid maps with a box size of 48 Å × 48 Å × 48 Å points and grid-point spacing of 0.375 Å were used. Each simulation was performed 10 times, yielding 10 docked conformations. Of note, conformations with the lowest energy were selected for the binding conformations between trametinib and MEK1.

### 3.3. MD Simulations

The GROMACS 5.1.4 package [34] with Gromos 53A6 force field was applied to describe the WT protein, three mutants (P124S, E203K, and A52V), and trametinib. The parameterization of trametinib was performed by the PRODRG2.5 server [35]. All complex systems were analyzed by MD simulations in a periodic boundary box with the SPC water model. To neutralize the systems, chloride and sodium ions were randomly added to the simulation box. In addition, energy minimization was performed through the steepest descent method, where the energy-minimized structure was allowed to reach an initial structure of equilibration. Subsequently, 100 ps of NVT (Berendsen temperature coupled with constant particle number, volume, and temperature) and 100 ps of NPT [36] (Parrinello–Rahman pressure coupled with constant particle number, pressure, and temperature) were performed to maintain the stability of the system (300 K, 1 bar). The coupling constants for temperature and pressure were set at 0.1 and 2.0 ps, respectively. Long-range electrostatic interactions were described using the particle mesh Ewald algorithm with an interpolation order of 4 and a grid spacing of 1.6 Å. Van der Waals interactions were calculated according to the cutoff value of 12 Å. All bond lengths were constrained using the LINear Constraint Solver (LINCS) algorithm [37]. After stabilizing all thermodynamic properties, the molecular system was simulated for 500 ns with a time interval of 2 fs, whereas the coordinates for all models were stored every 2 ps.

### 3.4. Protein Structure Network Analysis

The network parameters (for clusters, hubs, cliques, and communities) were utilized to analyze the residue interaction networks. The clusters and nodes (at least two) in the network were calculated by the MCODE algorithm and visualized in Cytoscape [38]. The hubs were defined as highly connected nodes in the network with at least four associated edges on the node. A k = *n* clique was a group of “*n*” nodes, in which each one was connected to every other node in the group. A community of k = *n* cliques indicated a collection of cliques sharing *n*-1 nodes among themselves. The network parameters for hubs, cliques, and communities were computed by the clique percolation method when implemented in CFinder software [37]. RINerator was used to generate edge attribute files. The protein structures used in protein network analysis were the representative structures from snapshots of the last 500 ns in the four systems.

### 3.5. Cross Correlation Analysis and Principal Component Analysis (PCA)

The cross-correlation matrix map suggested the motions for the most variance in the target atomic position when diagonalizing the covariance matrix of the atomic coordinates of the system. PCA was considered as an effective and useful tool for the identification of large-scale motions and the correlated movements of macromolecular biological systems. Of note, PCA has become prevalent for its wide application to protein systems by reducing or simplifying large and complicated data sets along trajectories generated by MD simulations [39].

### 3.6. MM-PBSA Calculations

The lowest energy of the two structures with the last conformation at 500 ns MD simulations was used as a starting point to calculate binding free energies. In addition, the binding free energies were calculated using molecular mechanics–Poisson–Boltzmann surface area (MM–PBSA) [40], which were calculated by the MM-PBSA method [41] in the GROMACS 5.1.4 package in our study. A total of 100 snapshots were evenly selected from the MD trajectory. The total binding energy (ΔG_bind_) was computed using the following equation:

ΔG_bind_ = G_complex_ − (G_protein_ + G_ligand_) (1)

where ΔG_bind_ represents the binding free energy between the protein and the ligand. ΔG_bind_ is the difference between the total free energy of the complex (G_complex_) and the sum of the free energy of the protein (G_protein_) and the ligand (G_ligand_). The binding energy is expressed as the combination of enthalpy and entropy terms:

ΔG_bind_ = ΔH − TΔS (2)

where TΔS refers to the entropic contribution to the free energy in a vacuum, in which T and S denote temperature and entropy, respectively. The enthalpy of binding can be further decomposed into protein–ligand and solvation free-energy contributions.

ΔH = E_MM_ + G_solvation_ (3)

where E_MM_ is the molecular mechanics energy of the molecule expressed as the sum of the internal energy of the molecule plus electrostatic and van der Waals energies. The solvation free energy is expressed as polar and non-polar contributions to the solvation energy:

E_MM_ = E_vdw_ + E_ele_ (4)

G_solvation_ = G_polar_ + G_nonpolar_ (5)

G_nonpolar_ is calculated from the solvent-accessible surface area (SASA):

G_nonpolar_ = γSASA + b (6)

where γ = 0.0072 kcal/mol/Å, and b = 0 kcal/mol.

### 3.7. Steered Molecular Dynamics Simulations

To reveal the effects of mutation on the dissociation process of trametinib from MEK1, the center of mass of trametinib was forced to pull out along a predefined direction using the GROMACS 5.1.4 package. The force field was the same as the conventional dynamic simulation. The direction of pulling was defined by two points. To be specific, the first point was the location of the active site, and the second point was the center of trametinib. In our study, constant velocity ensemble SMD simulations were performed. The spring did not move in a time-accessible SMD simulation, so a constant of 0.5 kcal· mol^−1^ ·A^−2^ was adopted to stretch the imaginary atom from the central mass of the SMD atom with constant velocity. The four ligand–receptor systems were performed in 10 ns SMD simulation. Three replications for two complexes were simulated in this study.

## 4. Conclusions

MEK occupies a crucial downstream signaling node of RAS, RAF and ERK protein. Thus, MEK has long been the target of drug discovery. However, the binding mode of trametinib to WT and mutant MEK1 and the dissociation of trametinib from them remain currently unknown to date. Therefore, in this study, molecular docking, MD simulations, and SMD simulations were performed to investigate the conformational changes for the WT MEK1 and three mutants (A52V, P124S, and E203K), as well as the dissociation of trametinib from the WT MEK1 and mutants. According to the outcomes from the MD study, we concluded that the large movements were concentrated on activation segments of the E203K and P124S MEK1, and the curvature of the entrance of allosteric channel in active type was larger than that in nonactive type. All these results should be responsible for the decreased interactions between MEK1 and trametinib. Moreover, the SMD study revealed that the dissociation of trametinib from non-active MEK1 was much easier than that from active MEK1, which was consistent with the conclusion of MD study. Collectively, our study provided a rational clue for designing novel MEK inhibitors for anti-cancer therapy associated with RAS-RAF-MEK-ERK signaling pathway.

## Figures and Tables

**Table 1:** The probability for residues of the helix during MD simulations.

System	Probability for α_Helix (%)	Probability for 3_10__Helix (%)
	N214-I216	P193-N195
WT	54.6	62.0
A52V	94.7	79.6
E203K	2.8	17.1
P124S	8.8	44.2

**Table 2:** The probability of hydrogen bonds formation between protein and trametinib.

Hydrogen Bonds		WT (%)	A52V (%)	P124S (%)	E203K (%)
Donor	Accepter				
Gly210:N	MOL:F34	81.8 ± 0.4	81.0 ± 0.4	23.9 ± 0.4	67.4 ± 0.5
Gly213:N	MOL:C10	40.8 ± 0.5	44.5 ± 0.5	--	--
Val211:N	MOL:O8	84.0 ± 0.4	80.9 ± 0.4	21.1 ± 0.4	79.6 ± 0.4
Ser212:N	MOL:O8	91.4 ± 0.3	81.4 ± 0.4	23.7 ± 0.4	--
Ser212:OG	MOL:O8	58.4 ± 0.5	80.5 ± 0.4	45.9 ± 0.5	22.2 ± 0.4
Ser212:CB	MOL:O8	77.0 ± 0.4	62.5 ± 0.5	37.1 ± 0.5	--
Ser212:OG	MOL:C7	81.9 ± 0.4	73.3 ± 0.4	33.4 ± 0.5	58.6 ± 0.5
Lys97:NZ	MOL:O12	71.6 ± 0.5	86.8 ± 0.3	65.3 ± 0.5	46.3 ± 0.5
Phe209:N	MOL:F34	37.9 ± 0.5	47.4 ± 0.5	26.0 ± 0.4	--
Lys97:CD	MOL:O12	72.6 ± 0.4	40.8 ± 0.5	20.4 ± 0.4	39.3 ± 0.5
Lys97:CE	MOL:O12	66.6 ± 0.5	32.7 ± 0.5	23.4 ± 0.4	37.6 ± 0.5
Gly210:CA	MOL:F34	73.1 ± 0.4	66.9 ± 0.5	--	41.9 ± 0.5
MOL:C35	Asp208:N	31.6 ± 0.5	20.8 ± 0.4	--	--
Val211:N	MOL:F34	--	36.1 ± 0.5	--	--
Ser212:CA	MOL:O8	81.9 ± 0.4	73.5 ± 0.4	--	28.9 ± 0.5
MOL:N22	His100:O	35.7 ± 0.5	24.5 ± 0.4	--	--
Asp208:N	MOL:C36	36.3 ± 0.5	36.6 ± 0.5	--	--

--: Probability values less than 20% are omitted.

**Table 3:** The MM-PBSA results. All energy values are given in kcal/mol.

System	WT	A52V	P124S	E203K
ΔE_ele_	−85.0 ± 10.4	−87.9 ± 8.3	−70.0 ± 7.5	−69.9 ± 6.2
ΔE_vdw_	−54.6 ± 4.9	−51.7 ± 4.7	−41.0 ± 3.0	−43.7 ± 3.9
ΔG_np_	−5.6 ± 2.0	−5.4 ± 1.6	−4.8 ± 2.2	−5.0 ± 1.7
ΔG_pb_	90.1 ± 13.6	93.2 ± 12.1	75.1 ± 10.8	76.5 ± 15.3
TΔS	−60.2 ± 5.5	−59.1 ± 4.9	−45.9 ± 3.4	−48.7 ± 3.2
ΔG_bind_	−55.0 ± 3.9	−53.9 ± 2.5	−40.7 ± 2.1	−42.2 ± 3.3

## Supplementary Materials

Supplementary materials can be found at https://www.mdpi.com/1422-0067/21/6/2167/s1.
