# Supplementary material for: Computational Study on the Effect of Inactivating/Activating Mutations on the Inhibition of MEK1 by Trametinib
Source: Int J Mol Sci. 2020 Mar 21;21(6):2167. doi: 10.3390/ijms21062167 (PMC7139317; doi:10.3390/ijms21062167)

Figure S1. Dynamic changes of the secondary structure profile for (A) WT, (B) A52V mutant, (C) E203K mutant and (D) P124S mutant MEK-1 throughout the simulation. The colored bar represented different secondary structures as follows: coil (C), π-helix (I), helix (G), α-helix (H), β-Bridge (B), β-bugle (E), turn (T).

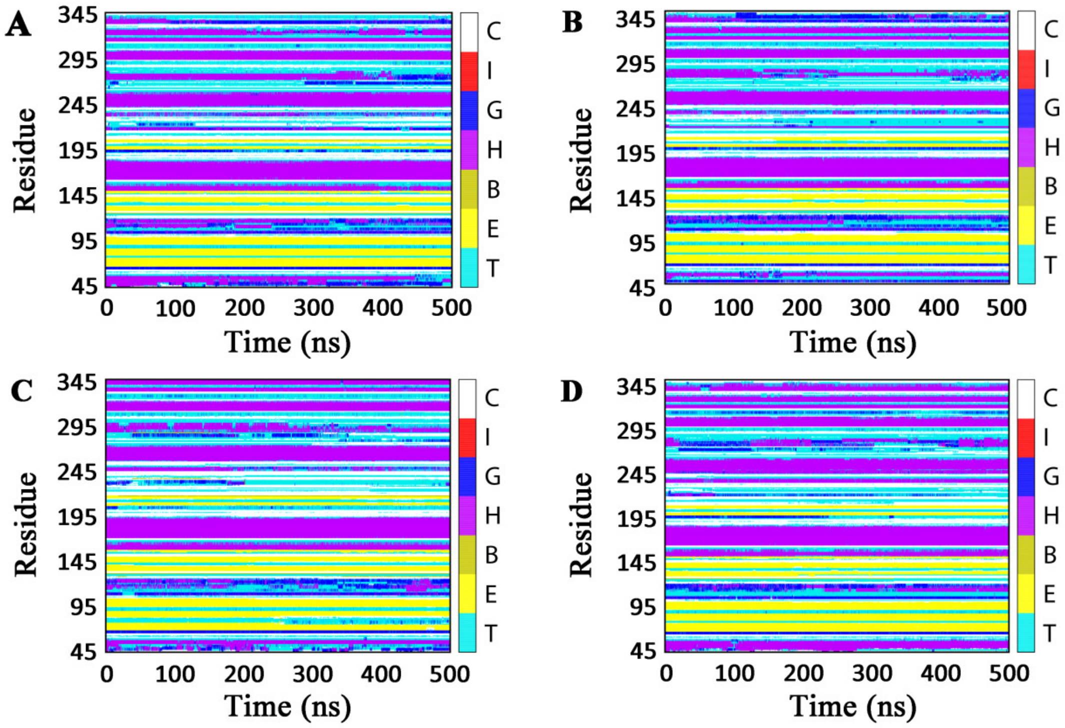

Figure S2 Typical force profiles of ligands pulled out of the MEK1 pocket along the unbinding pathway.

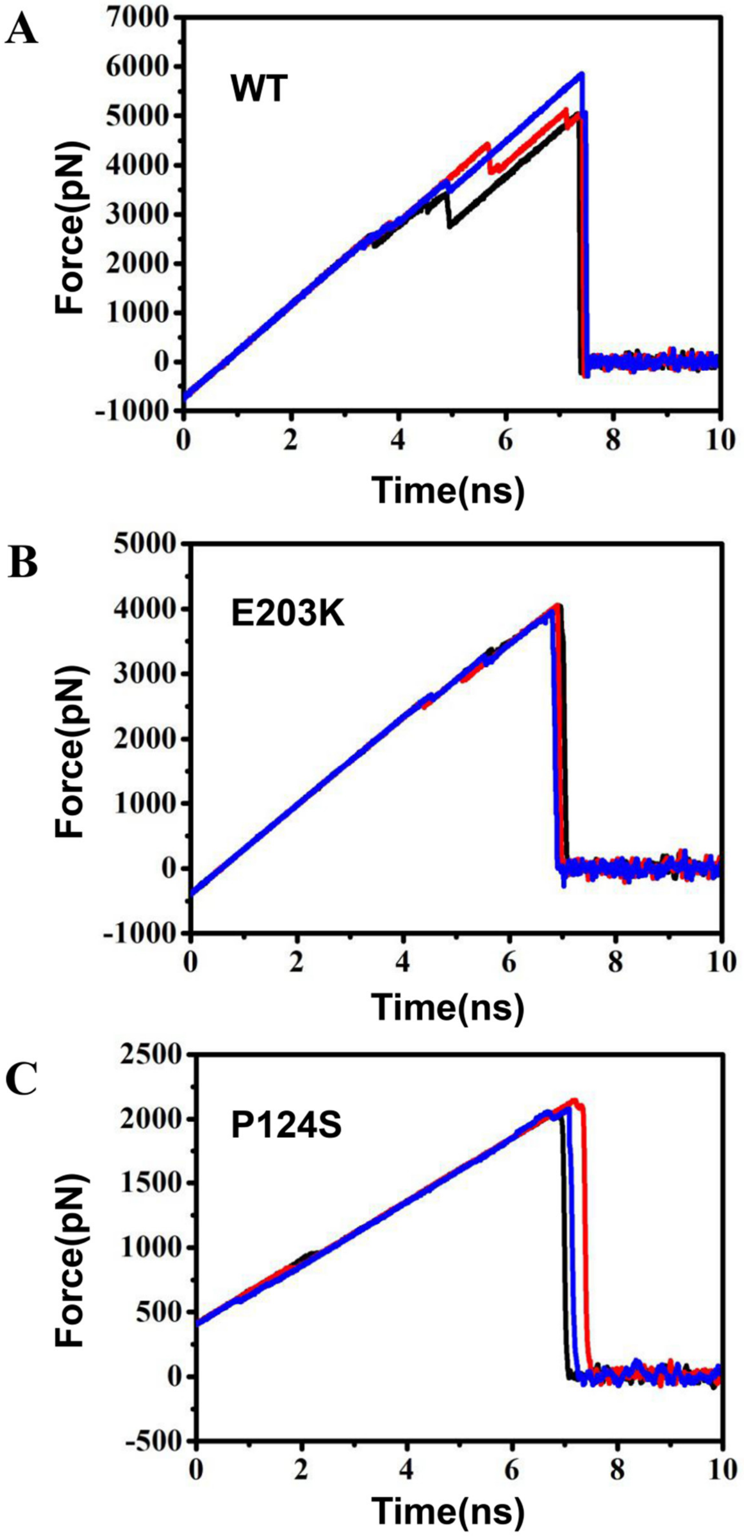

Supplement: Supplementary file 1 [file ijms-21-02167-s001.pdf]
